# Evaluation of Fundus Blood Flow in Normal Individuals and Patients with Internal Carotid Artery Obstruction Using Laser Speckle Flowgraphy

**DOI:** 10.1371/journal.pone.0169596

**Published:** 2017-01-05

**Authors:** Yoichiro Shinohara, Tomoyuki Kashima, Hideo Akiyama, Yukitoshi Shimoda, Danjie Li, Shoji Kishi

**Affiliations:** Department of Ophthalmology, Gunma University School of Medicine, Maebashi, Gunma, Japan; Bascom Palmer Eye Institute, UNITED STATES

## Abstract

**Purpose:**

We investigated whether laser speckle flowgraphy (LSFG) results are comparable in both eyes and whether it is useful in the diagnosis of disparity in ocular ischemic syndrome (OIS) patients.

**Methods:**

We compared the mean blur rate (MBR) value for various fundus regions in both eyes of 41 healthy subjects and 15 internal carotid artery occlusion (ICAO) cases. We calculated the standard value of the Laterality Index (LI), which was the MBR comparison of both eyes in each of the regions, in the control subjects. We then investigated the correlation between both eyes for the LIs in the entire fundus, the degree of ICAO and visual function.

**Results:**

The disparity of the LIs in both eyes was least in the entire area of the fundus in control subjects and there was a significant correlation between both eyes of the 41 healthy individuals (P = 0.019). Significant correlations were found for the LI, visual acuity and degree of ICAO. The specificity and sensitivity of LI in the entire area was 93.8% and 100%, respectively.

**Conclusions:**

LSFG revealed normal individuals have symmetrical fundus blood flow. LSFG could detect OIS and might be a useful tool for detecting disparities in fundus blood flow.

## Introduction

Ocular ischemic syndrome (OIS), which accompanies ischemic ophthalmopathy, is caused by a decrease in ocular blood flow that is the direct result of diminished ophthalmic artery blood pressure due to occluded regions in the internal carotid artery (ICA).[1] In cases of chronic advanced OIS, both subjective symptoms (e.g., visual loss or pain) and objective findings (e.g., retinal hemorrhages or rubeosis iridis) are often unclear.[2, 3] In addition, sometimes OIS cannot be immediately diagnosed because there is a lack of ischemic findings in the posterior fundus due to these changes being hidden by retinal and/or vitreous hemorrhages, or by the fibrosis and panretinal photocoagulation associated with diabetic retinopathy.[4]

In OIS patients, severe carotid artery disease can cause transient visual loss as a result of choroidal hypoperfusion[2] or retinal or optic nerve infarction. [5] Brown et al. reported finding decreased visual acuity (VA) in 91% of the affected eyes, with the visual loss occurring chronically over a period of weeks to months in 67% of these eyes.[6] Mizener et al. reported that the first presentation of visual loss in OIS patients occurred suddenly in 41% of the eyes, was gradual in 28%, and in 21%, the eyes exhibited no visual loss. The study also reported that VA at the initial visit in the OIS eyes was less than or equal to 20/400 in 64% of the patients.[7] In contrast, 10–15% of the OIS patients initially presented with transient visual blindness. [8] Many of these patients stated that their vision sometimes felt patchy or that they noticed sectorial loss. These conditions were found to be caused by either emboli or vasospasms of the carotid artery or ophthalmic artery. [9–11] However, there have been no reports on the relationship among visual symptoms and choroidal blood flow.

Laser speckle flowgraphy (LSFG) can be used to non-invasively determine the blood flow of the fundus and display it as a two-dimensional false color map or moving image. The latest LSFG model is able to measure the fundus blood flow at a 30° angle, which makes it possible to simultaneously observe the optic disc, macular area and major retinal vessels. [12] Using the experimental branch retinal artery occlusion model of monkeys, Isono et al. additionally determined that 92% of the LSFG analysis value actually reflected choroidal blood flow, while the false color map that was created using these data resembled the results of Indocyanine Green Angiography (IA). [13]

In the current study, we investigated various fundus areas in both healthy individuals and in internal carotid artery occlusion (ICAO) patients. One of our major findings was that we showed it was possible to non-invasively obtain blood flow maps with false color that definitively determined decreases in the blood flow throughout the entire fundus in OIS patients with visual disturbances.

## Methods

### Ethics Statement

This study was approved by the institutional review board of Gunma University Hospital (No. 160051). Written informed consent was obtained from all participants. The anonymity of the patients was kept.

### LSFG-Wide Measurements of Fundus Blood Flow

In the previous LSFG model, either the normalized blur rate (BR) or the relative velocity of the erythrocytes were used as an index that represented the blood flow volume. The device utilizes a diode laser (wavelength: 830 nm) to illuminate the moving erythrocytes, with the reflected light generating a speckle pattern that is then used to calculate the blood flow values. The LSFG-wide device utilizes the mean blur rate (MBR), which is two times the squared BR, as the indicator of the relative velocity of the erythrocytes. [12]

**Fig 1:**
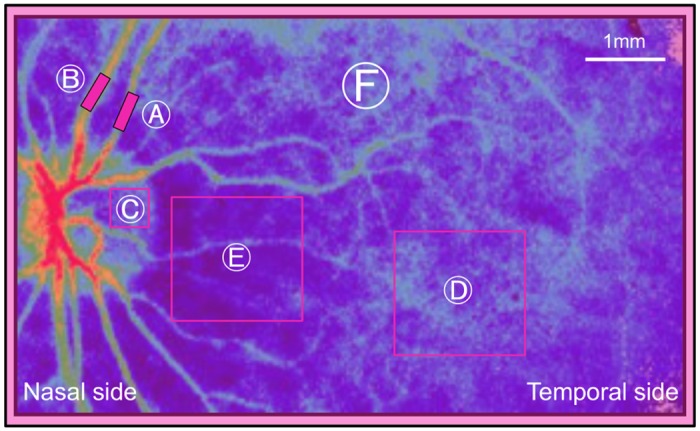
Regions in which the mean blur ratio were measured. A indicates the retinal artery; B, the retinal vein; C, the temporal sector of the disc; D, the macular area; E, the peripapillary area; and F, the entire image.

In all cases, LSFG was performed in both eyes, with the subjects in a sitting posture. For the examination of each part, we used an arbitrary rectangular band on the MBR map. The retinal vessel MBR value was measured between the first branch and the papilla without inclusion of the non-vessel tissue. Retinal arteries and veins were identified based on fundus photographs. For the MBR value in the marginal area of the optic disc, measurements were performed on the temporal side of the disc. We set the arbitrary region of interest for each patient to avoid the main retinal vessels. Since the MBR value from the macular and peripapillary areas can be influenced by large choroidal vessels, we measured a square with a side length of 1 disc diameter to reduce the fluctuation as much as possible. The right and left differences were compared for each part of the retinal artery/vein, marginal area of the optic disc, macular area, temporal peripapillary area, and all other area within the images (Fig 1).

However, it should be noted that there are potential hazards to human eyes when using this apparatus and these must be discussed with the patients prior to performing any measurements. According to the guidelines of the American National Standards Institute, the maximum permissible retinal exposure when viewing a diffuse reflection of a diode laser is 460 mW/cm^2^ over a 10-second period. In the current study, the maximal exposure in each of the subject's retinas when using the present apparatus was approximately 90 mW/cm^2^ with a total exposure time of 4 seconds. These values were far below the stated permissible limits. [14–16]

### Control Group

LSFG was used to examine 41 healthy individuals that had no previous history of ocular or circular disease (82 eyes; male: female ratio = 14:27; average age = 44.1±17.8 years old). MBR differences in the right and left eyes of all of the subjects were investigated in each part of the retinal artery/vein area, marginal region of the optic disc, macular region, and the peripapillary region.

### Patients Group

A total of 15 patients were diagnosed with unilateral internal ICAO in the Department of Neurosurgery in Gunma University between April of 2006 and November of 2010 (male: female ratio = 11:4; age range: 49 to 80 years old; average age = 66.1±7.9 years old). Magnetic Resonance Angiography, Digital Subtraction Angiography, or cervical ultrasonography examinations detected the area of obstruction and calculated the degree of ICA occlusion in all cases. There was no hazy media such as corneal edema, severe senile cataract or vitreous hemorrhage, noted in any of the 15 cases. The patients who had central/branch retinal artery/vein occlusion and macular edema were excluded. The blood pressure and intraocular pressure were measured. Continuous visual disturbance was noted in 7 of the 15 cases and these patients were classified as Group VD (visual disturbance). In 2 of these 7 cases, diabetic retinopathy and neovascular glaucoma were observed, with the intraocular pressures rising to 26 mmHg and 30 mmHg, respectively. In addition, 4 of the 15 cases had an awareness of amaurosis fugax symptoms, while the remaining 4 cases had no awareness of any visual difficulties. These 8 cases were classified as Group non-VD.

### Bilateral Comparison and Calculation of Standard Limit

Aizawa et al. used the affected/fellow eye MBR ratio to assay the hemodynamics of rhegmatogenous retinal detachment patients. [17] We also defined the laterality index (LI) as the ratio of the MBR of the right to left eyes in normal controls and that of the affected to fellow eye in patients with OIS. The LI was calculated in each region of the ocular fundus and the entire image. We obtained a standard LI value using the following formula: standard LI value = mean LI ± 2×standard deviation (SD).

When analyzing the patient group, LI was calculated using the following formula:Laterality Index (LI) = MBR value of the affected eye / MBR value of the fellow eye.

### Specificity and Sensitivity

Using data from the 15 ICAO patients and 41 controls, the specificity and sensitivity of LI for the entire area were calculated to evaluate the application for clinical use.

### Statistical Analysis

Data were presented as mean ± SD. Comparison of the differences between both eyes in the control group was performed using Student’s t-test. Pearson’s correlation coefficient test and Spearman’s correlation coefficient by rank test were calculated to determine the association between the LI, VA, and the degree of ICA occlusion. Statistical significance was defined as P<0.05. All statistical analyses were carried out using SPSS for Windows (SPSS Japan Inc., Tokyo, Japan).

## Results

### Comparison of Both Eyes in the Control Group

**Fig 2:**
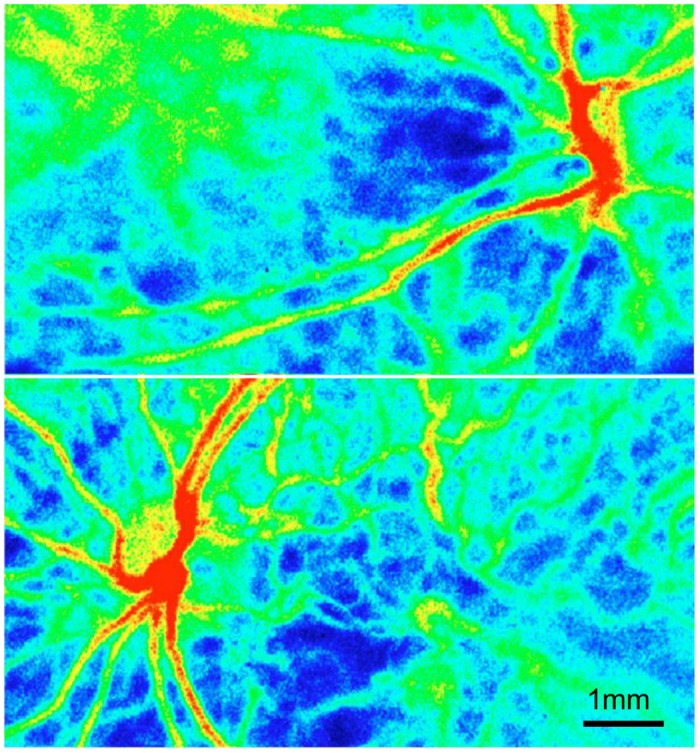
False color maps of bilateral Laser Speckle Flowgraphy in both eyes of a normal subject. Top, right eye; bottom, left eye. The right and left differences in the normal subjects was not significant in the blood flow.

**Fig 3:**
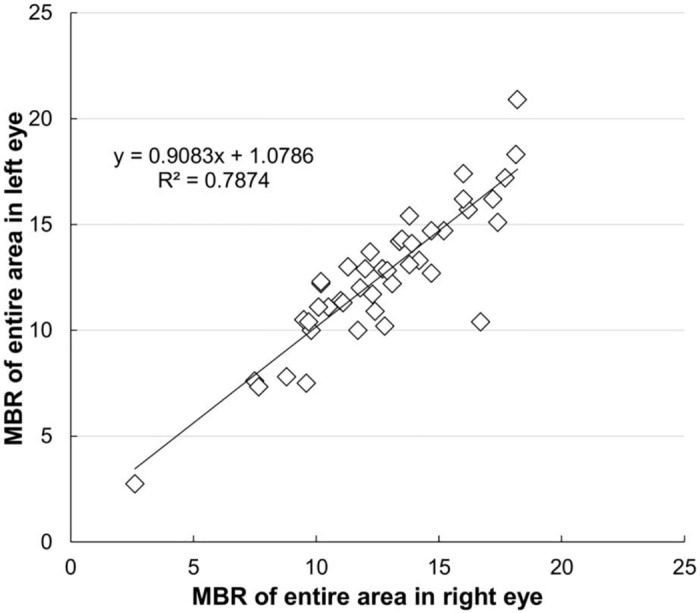
Bilateral comparison of the mean blur ratios in the entire image in normal individuals. Correlation coefficient of the linear fit r = 0.8594 (P = 0.019).

Among the normal individuals, the false color maps created for both eyes revealed almost identical images (Fig 2). As shown in Table 1 and S1 Table, the mean MBR value in the right eye of the healthy individuals was 31.3±10.0 in the retinal arteries, 35.6±6.1 in the retinal veins, 12.7±3.0 in the marginal region of the optic disc, 12.3±3.8 in the macular region, 8.7±2.7 in the peripapillary region and 12.9±2.6 in the entire area. For the left eye, the values for the same areas were 29.1±9.1, 33.2±9.7, 12.0±2.6, 12.0±4.0, 9.0±3.0, and 12.8±2.7, respectively. When the areas between both eyes were compared, no significant differences were found for any of the regions (Student’s *t*-test). Based on these results, we calculated the standard value of the LI to be between 0.29 and 2.00 for the retinal artery, between 0.48 and 1.77 for the retinal vein, between 0.61 and 1.56 for the marginal area of the optic disc, between 0.39 and 1.73 for the macular region, between 0.42 and 1.59 for the peripapillary area and between 0.74 and 1.29 for the entire area. We defined the abovementioned standard value ranges of the LI as “normal LI”. Likewise, values out of the normal LI range were defined as “abnormal LI”. For the control group, the standard deviation for the LI in the entire area was found to be the smallest among all of the measured areas (Fig 3). In addition, there was a significant correlation noted for the MBR in the entire area of both eyes (P = 0.019). Based on this, the LI in the entire area is probably the best parameter to use when comparing both eyes.

**Table 1:** Summary of MBR values from 41 healthy individuals.

Area for measure	Mean MBR value		Mean LI	Standard deviation	Standard LI value	
	Right eye	Left eye			Lower border	Upper border
Retinal artery	31.3	29.1	1.15	0.42	0.29	2.00
Retinal vein	35.6	33.2	1.12	0.32	0.48	1.77
Margin of disc	12.7	12.0	1.08	0.24	0.61	1.56
macula	12.3	12.0	1.06	0.33	0.39	1.73
Para-papilla	8.7	9.0	1.00	0.29	0.42	1.59
Entire area	12.9	12.8	1.02	0.14	0.74	1.29

### Comparison of Both Eyes in the Patient Group

**Fig 4:**
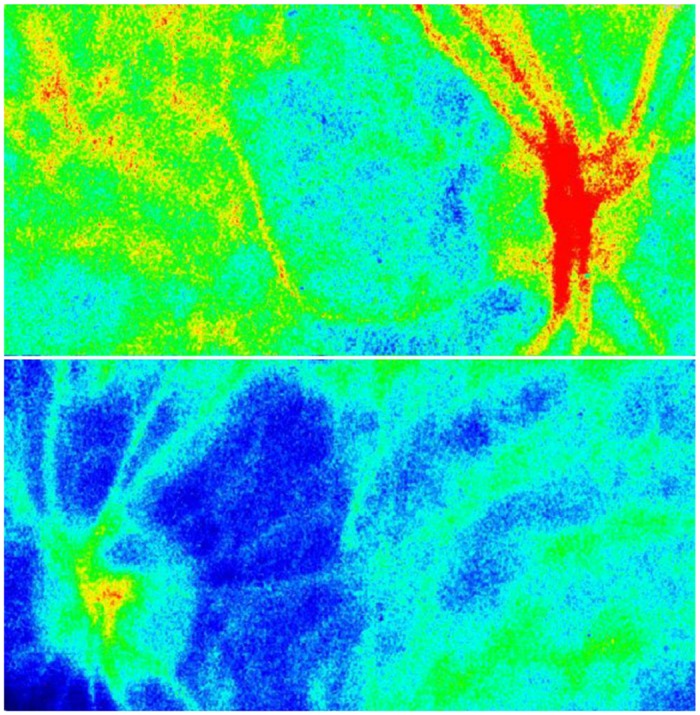
Case 1. The false color maps of a patient with ocular ischemic syndrome. Top, right eye; bottom, left eye. Although the color map of the right eye is normal, that of the left eye shows that the choroidal vasculature has disappeared and that there are faint retinal vessels present, suggesting decreased retinal and choroidal blood flows.

Table 2 showed the OIS patient characteristics. All 7 patients of the Group VD exhibited an abnormal LI in the entire area. The extent of the differences between the impaired eye and the fellow eye was so great for the false color maps that we were able to judge the decreased blood flow by simply viewing these maps (Fig 4). In Cases 1, 4, 5, 6 and 7, the LI was found to be below the normal range in each of the measured areas. The impaired eye of Case 2 exhibited an abnormal LI in all areas except within the margin of the disc. In Case 3, while the impaired eye exhibited an abnormal LI for the retinal artery/vein and peripapillary choroid, a normal LI was observed in the macular area and in the margin of the optic disc. In case 7, the MBR in each region were immeasurable because the blood flow was severely reduced in each region. In patients with transient blindness, the LI for all of the areas were within the normal range except for the entire area in Cases 8 and 10. In patients with no symptoms, the impaired eye of Case 12 exhibited a low LI in both the peripapillar and entire area, while all of the other cases had normal LIs in each of the measured areas.

**Table 2:** Patient MBR values for each of the comparison areas.

Case no.	Symptom	R/L	ICA Occlusion (%)	VA of Affected eye	LI value					
					Retinal artery	Retinal vein	Margin of disc	Macula	Para-papilla	Entire area
1	VD	L	100	20/400	0.25*	0.30*	0.52	0.52*	0.47	0.46*
2	VD	L	100	20/400	0.17*	0.13*	0.77	0.73	0.50	0.38*
3	VD	R	99	20/400	0.34	0.35*	0.79	0.62	0.59	0.57*
4	VD	L	Unknown	20/200	0.35	0.43*	0.54*	0.39*	0.47	0.56*
5	VD	R	100	20/60	0.12*	0.29*	0.47*	0.26*	0.23*	0.28*
6	VD	L	100	20/400	0.12*	0.11*	0.43*	0.20*	0.35*	0.26*
7	VD	R	100	20/400	Immeasurable	Immeasurable	Immeasurable	Immeasurable	Immeasurable	0.13*
8	TB	R	50	20/20	0.93	1.00	0.73	0.65	0.72	0.73*
9	TB	L	84	20/30	0.78	0.89	1.23	1.05	1.05	0.99
10	TB	R	50	20/30	0.78	0.79	1.03	1.03	1.24	1.36*
11	TB	R	84	20/15	1.17	1.27	1.00	1.29	0.97	0.94
12	N	R	50	20/20	0.89	1.14	0.97	0.78	0.54	0.78
13	N	L	84	20/30	0.79	0.79	1.12	1.38	1.25	1.13
14	N	L	50	20/15	1.12	1.00	0.83	1.01	1.09	0.99
15	N	L	90	20/15	1.15	1.05	1.23	0.80	1.04	0.95

VD, visual disturbance; TB, transient blindness; N, no subjective symptoms;

* = out of the standard LI value.

**Fig 5:**
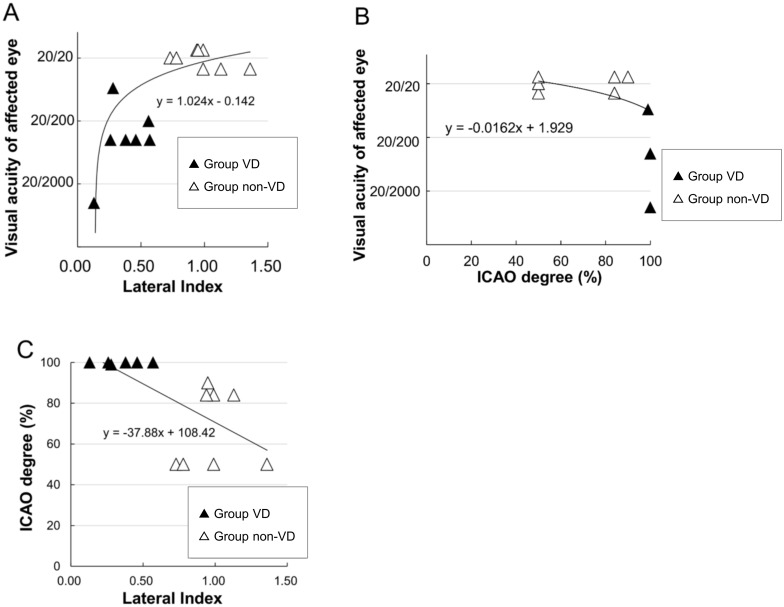
Correlation of the laterality index (LI) and visual acuity (VA) in patients with internal carotid artery obstruction (ICAO). (A) Correlation of the LI and VA in affected eyes. Comparison of group VD and the non-VD group. A significant correlation is seen between the LI and the ICAO severity (P = 0.0068). (B) A significant correlation is seen between the ICAO severity and the VA (P = 0.0041). (C) A significant correlation is seen between the LI and the ICAO severity (P = 0.0074).

We also investigated the correlation between the VA, the degree of ICA occlusion, and the LI in the entire area (Fig 5A–5C). The patients of Group non-VD are integrated in these figures. Since Case 4 was not analyzed by neurosurgeons, this case was excluded from the analysis of the degree of ICAO present. A positive correlation was found for the LI and VA (correlation coefficient = 0.7286) (P = 0.0068). In addition, the degree of ICAO and VA, along with the LI and degree of ICAO were shown to be inversely correlated (correlation coefficient = -0.6912 and -0.6747) (P = 0.0041 and 0.0074).

### Evaluated Specificity and Sensitivity

Using patient and control data, patients were divided into 4 groups according to both LI results and clinical diagnosis of OIS. Seven patients had OIS with an abnormal LI for the entire fundus area, three did not have OIS with an abnormal LI for the entire fundus area, 46 did not have OIS with a normal LI for the entire fundus area and none had OIS with a normal LI for the entire fundus area. Therefore, the calculated specificity and sensitivity of the LI for the entire fundus area was 93.9% and 100%, respectively.

## Discussion

In the current study, we demonstrated a significant correlation between the eyes of the healthy subjects. A previous study showed that MBR showed large variations in arteries, veins and choroidal areas. [18] Similarly, large variations in the LI trends of these areas were observed in the current study. However, the LI of the entire image had the lowest standard deviation and was close to 1.0, indicating that measurements over the entire image are the most reliable parameter for evaluating disparities in ocular blood flow in both eyes of a patient. Furthermore, in the non-invasively obtained blood flow maps with false color, a decreased blood flow was noted throughout the entire fundus in the OIS patients with visual disturbance. Our results indicated that the extent of the visual disturbance in these OIS patients was proportional to the magnitude of the choroidal blood flow decrease. Thus, there may be a correlation between visual symptoms and choroidal blood flow. To the best of our knowledge, this is the first report to document the non-invasive capabilities and the ease of use of LSFG-wide in diagnosing OIS especially associated with unilateral ICAO.

The most conventional and accessible diagnostic methods for OIS are commonly held to be Fluoresceine Angiography (FA) [2] and IA [19]. Utsugi et al. reported that the arm-to-choroid circulation time and the choroidal circulation time in OIS eyes was prolonged as compared to normal eyes. [19] IA is an adequate tool for diagnosis of OIS eyes as compared to FA, as observation of the choroidal blood flow and its disturbance can be performed quite simply. However, performing IA in all suspected OIS patients can sometimes be unfeasible due to the presence of hypertension, diabetic mellitus or cardiac diseases which may lead to fatalities in these patients. [20, 21]

Several authors have recently reported that there is autoregulation of the choroidal vessels. [22–25] In a study by Riva et al., they reported finding that a mild decrease of the perfusion pressure barely affected the choroidal blood flow. [25] However, when there was a severe decrease in the perfusion pressure, a rapid decrease in the choroidal blood flow occurred. In the current study, patients in Group VD had a significantly lower LI in the entire area as compared to the Group non-VD. Moreover, a significant correlation was noted for ICAO, LI and VA. Thus, these results demonstrate that only severe ICAO can lead to a decrease of the LI, which in essence means there was a decrease in the choroidal blood flow. This suggests that a choroidal blood decrease may also play an important role in visual disturbance of OIS patients. Furthermore, it should be noted that once neovascular glaucoma occurs, the already decreased ocular blood flow can be easily disturbed by a high ocular pressure, which further leads to a poor prognosis. [8] Earlier diagnosis of OIS with the use of LI could prevent the occurrence of neovascular glaucoma, even at severe ICAO stage.

Since the LSFG can measure blood flow in a small area of the fundus, it has been mainly used to obtain blood flow data for use in basic research. And even despite the fact that LSFG has on occasion been used to analyze the blood flow of the optic disc in glaucomatous eyes, there have been few reports that have used LSFG as a clinical diagnostic tool. [12, 26] A previous report showed that LSFG could be used to monitor changes in the ocular blood flow caused by nonischemic type central retinal vein occlusion. [27] Our results also suggested that LSFG-wide could be used for non-invasive observation of the circulatory dynamics in the posterior region, including the optic disc and macular lesions. Despite significant disparity in MBR between left and right eyes, it may be difficult to diagnose OIS with LSFG alone. However, the results of this study indicated a correlation between LI determined using LSFG-wide, degree of ICAO, and severity of OIS. Thus, LSFG may be useful in facilitating the diagnosis of OIS in patients with undefined unilateral visual disturbance.

## Supporting Information

S1 Table MBR values for each of the comparison areas in both eyes of normal individuals. Abbreviations as in Table 1.
(XLSX)
